# Parathyroidectomy for primary hyperparathyroidism: effect on quality of life after 3 years – a prospective cohort study

**DOI:** 10.1097/JS9.0000000000000282

**Published:** 2023-03-24

**Authors:** Samuel Frey, Bastien Perrot, Cécile Caillard, Maëlle Le Bras, Maxime Gérard, Claire Blanchard, Bertrand Cariou, Matthieu Wargny, Eric Mirallié

**Affiliations:** a Nantes Université, CHU Nantes, Chirurgie Cancérologique, Digestive et Endocrinienne, Institut des Maladies de l’Appareil Digestif; b Nantes Université, CHU Nantes, CNRS, INSERM, l’institut du thorax; c Plateforme de Méthodologie et de Biostatistique – DRCi – CHU de Nantes; d Nantes Université, CHU Nantes, Service d’Endocrinologie, Diabétologie et Nutrition, l’institut du thorax, Nantes; e CHU de Nantes, INSERM, Pôle Hospitalo-Universitaire: Santé Publique, Santé au Travail, Pharmacie, Stérilisation, Clinique des Données, Paris, France

**Keywords:** parathyroidectomy, primary hyperparathyroidism, quality of life

## Abstract

The impact of parathyroidectomy (PTX) for primary hyperparathyroidism (PHPT) on long-term quality of life (QoL) remains controversial. The study evaluated QoL changes 1 and 3 years after PTX.

**Materials and Methods:**

Patients undergoing PTX for PHPT between 2016 and 2022 (*n*=329) were enrolled in this monocentric, prospective cohort study. QoL was evaluated using the SF-36 questionnaire before, 1 year, and 3 years after PTX and compared with an age-matched and sex-matched French reference population. Only patients with 1-year and 3-year follow-up and complete evaluation (serum calcium, phosphorus, parathyroid hormone) were included.

**Results:**

A total of 159 patients were included (mean age: 62.6±12.7 years, 79.2% females). Mean serum calcium (2.66±0.20 mmol/l) and median parathyroid hormone (96.4 [76.9−126.4] pg/ml) levels improved significantly after PTX. Before surgery, PHPT patients had impaired physical (44.6±8.9 vs. 47.6±6.8 in the reference population, *P*<0.001) and mental (42.3±10.9 vs. 48.9±6.8, *P*<0.001) component scores. The mean physical component score increased significantly at 1 and 3 years and was no longer different from the reference population (ratio: 0.94±0.15 preoperatively vs. 0.99±0.15 at 3 years, *P*<0.01). The mean mental component score increased significantly at 1 and 3 years, but remained significantly lower than the reference population. Before surgery, a lower physical component score and younger age were significantly associated with a 3-year physical component score increase on multiple linear regression analysis.

**Conclusion:**

A significant improvement in QoL is associated with PTX for PHPT at 1 year and is sustained for at least 3 years after surgery.

## Introduction

Highlights

- The impact of parathyroidectomy for primary hyperparathyroidism on long-term quality of life remains controversial.
- Patients with primary hyperparathyroidism had an impaired quality of life (SF-36 questionnaire) in comparison with an age-matched and sex-matched general French population before surgery.
- Both SF-36 physical and mental component scores significantly improved 1 and 3 years after parathyroidectomy. The physical component score normalized in comparison with the general French population.
- Preoperatively, a lower physical component score and younger age were significantly associated with a 3-year physical component score increase.

Primary hyperparathyroidism (PHPT), one of the most frequent endocrine disorders after diabetes mellitus and thyroid diseases^1^, results from inappropriate parathyroid hormone (PTH) secretion by one or more abnormal parathyroid glands. PHPT typically leads to an elevated serum calcium level (SCa) with low phosphoremia. The classic features of PHPT include bone demineralization leading to fractures, nephrolithiasis, and renal insufficiency^2^. However, PHPT is currently more often diagnosed in Western countries in its mild form owing to early detection during biological screening with moderately high or normal SCa^3^. The clinical presentation of PHPT has therefore evolved from a symptomatic to an ‘asymptomatic’ disease – a term that is inaccurate since manifestations of PHPT are likely to occur^4^.

Indeed, neuropsychological symptoms, as well as a myriad of ‘nonspecific symptoms,’ many of which have been listed in the Pasieka Illness Questionnaire^5^, have been presented by ∼50% of the patients with mild PHPT^6^,^7^. Patients with PHPT, even in mild forms, have presented impaired health-related quality of life (HR-QoL)^8^. However, according to the recent guidelines, indications for parathyroidectomy (PTX) include age less than 50 years, SCa greater than 2.85 mmol/l, bone (osteoporosis defined by a *T*-score <−2.5 on one site and/or vertebral fractures) or renal disorders (creatinine clearance <60 ml/min or kidney stones) and elevated calciuria^9^. Nonclassic symptoms and impaired HR-QoL have not been recognized as surgical indications since the long-term effect of PTX on HR-QoL relies on inconsistent evidence^10^.

Indeed, while PTX has been associated with an improved HR-QoL in the short term after surgery in prospective noninterventional studies and randomized controlled trials^11^–^21^, long-term results are scarce and controversial^22^–^25^, and data concerning HR-QoL evaluation more than 1 year after surgery are strongly lacking^10^. Therefore, this study was conducted to add long-term data regarding HR-QoL, which could improve the quality of preoperative patients’ information.

The aim of this study was to evaluate the QoL change 3 years after PTX in a prospective cohort of patients with sporadic PHPT. HR-QoL was evaluated using the Short Form-36 (SF-36) questionnaire, and the results were compared with an age-matched and sex-matched reference French population. In order to assess if patients without surgical indication, as defined by the recent recommendations^9^, could benefit from surgery, a subgroup analysis regarding HR-QoL change was performed in this specific population.

## Methods

### Study population

The Cohorte Prospective Hyperparathyroïdie Primaire (‘prospective cohort with primary hyperparathyroidism patients,’ or CoHPT NCT05469087) is a monocentric prospective cohort designed to study the consequences of PTX for PHPT in which all consecutive patients diagnosed with sporadic PHPT in a French tertiary referral center were included. PHPT was defined as an elevated or inappropriate serum PTH level compared with an elevated (>2.60 mmol/l) SCa; or an elevated serum PTH level compared with a normal SCa^26^,^27^. Noninclusion criteria were age under 18, pregnancy, secondary or tertiary hyperparathyroidism, hypocalciuric hypercalcaemia, and/or multiple endocrine neoplasia.

All patients included in CoHPT between 31 March 2016 and 2 February 2022 were screened for the present analysis. The sample size was not calculated *a priori*. In addition to the standard preoperative evaluation, all patients included in CoHPT systematically underwent HR-QoL evaluation using the SF-36 questionnaire on inclusion, and 1 and 3 years after PTX. Patients who did not undergo PTX or reach the 3-year postoperative follow-up consultation when the study was conducted (operated <3 years before) or for whom critical data were missing (BMI, SCa, serum phosphorus, and PTH levels before and 1 year after PTX, SF-36 questionnaire before and 3 years after PTX) were excluded from the present analysis. This study is written in accordance with the STROCSS (Strengthening the reporting of cohort studies in surgery) guidelines^28^, Supplemental Digital Content 2, http://links.lww.com/JS9/A113.

A complementary analysis was performed specifically in patients with asymptomatic PHPT. Asymptomatic patients were defined as patients without preoperative surgical indications as defined by the last international guidelines^9^. Inclusion criteria for this subgroup were age at least 50, SCa 2.85 mmol/l or less, absence of osteoporosis (*T*-score ≥−2.5 at lumbar spine, total hip, femoral neck, and distal 1/3 radius on dual X-ray absorptiometry), absence of vertebral fractures less than 10 years, no history of symptomatic nephrolithiasis and estimated glomerular filtration rate [(eGFR) using CKD-EPI (Chronic Kidney Disease Epidemiology Collaboration) 2009 formulae] at least 60 ml/min. Since the present study began before the publication of the latest recommendations, which newly take into account 24-h urine calcium excretion per se as a surgical indication, this measurement has not been performed systematically in our patients. Calciuria has always been measured on spot urine samples as a diagnostic criterion. Therefore, patients with elevated 24-h urine calcium excretion were not excluded from this subanalysis.

All patients were operated on by two expert endocrine surgeons (performing >40 PTX/year). The surgical indications were in accordance with French guidelines^29^. A preoperative imaging workup, including neck ultrasonography and ^99m^Tc-MIBI scintigraphy, was systematically performed. Unilateral or bilateral neck exploration by cervicotomy was performed at the discretion of the surgeon. Cure was suspected intraoperatively when serum PTH level (measured at incision, during the gland dissection, and 10 and 20 min after the excision) decreased by more than 50% and reached a normal value 10 min after the gland resection. Cure was defined as normocalcemia (SCa <2.60 mmol/l) 6 months after PTX or, if not available, at the 1-year consultation.

### Outcomes

The primary outcome was HR-QoL change 3 years after PTX using the SF-36 questionnaire. Secondary outcomes were HR-QoL change 1 year after PTX; the association between preoperative variables and an improvement in post-PTX HR-QoL; and HR-QoL change specifically in the asymptomatic PHPT group.

### HR-QoL assessment

HR-QoL was assessed preoperatively and during the 1-year and 3-year consultations using the SF-36 questionnaire^30^ in its validated French version^31^.

Briefly, the SF-36 questionnaire consists of 36 items used to establish eight-dimension scores related to physical HR-QoL (physical functioning, vitality, bodily pain, role limitation due to physical problems) and mental HR-QoL (mental health, general health perception, social functioning, role limitations due to emotional problems) ranged from 0 to 100. The higher the score, the better the HR-QoL. Composite scores, namely physical and mental composite scores (PCS and MCS, respectively), were calculated by using these subscores and summarize these results.

Each SF-36 score of the included patients was compared with the score of the general French population using a normative score ratio according to age and sex^32^. For each dimension of the SF-36, the normative score ratio was computed as the ratio of the patient’s score to the average score of the general French population of the same age and sex. Score ratios lower than 1 indicate worse HR-QoL compared with the age-matched and sex-matched general population. Score ratios were computed for the preoperative, 1-year and 3-year consultations and the longitudinal changes in HR-QoL were expressed as the differences in score ratios across the three measurement times.

### Preoperative patient evaluation

Bone mineral density was measured before surgery using dual X-ray absorptiometry at the lumbar spine, left total hip, femoral neck, and 1/3 distal radius. A *T*-score less than −2.5 at a minimum of 1 site-defined osteoporosis. History of fracture less than 10 years and symptomatic nephrolithiasis were systematically searched. Patients were asked if they felt fatigued or depressed since they have been reported to be frequent nonclassical PHPT symptoms^6^,^33^. Comorbidities were recorded and used to calculate the cumulative illness rating scale (CIRS)^34^.

### Biological measurements

All biochemical measurements were performed in the accredited biochemical laboratory of CHU Nantes. Blood samples were taken after 12 h of fasting. Samples were analyzed on a Cobas 6000 Ce Analyzer (Roche, Meylan, France). SCa and urine calcium were measured via the NM-BAPTA method, serum phosphorus with the molybdate method, and serum creatinine using the enzymatic method. eGFR was calculated using the CKD-EPI equation. PTH and vitamin D were obtained using electrochemiluminescence immunoassays.

### Ethical approval

The CoHPT cohort is registered at CHU Nantes (No. 2015-031) in accordance with the French National Commission for Data Protection and Liberties (CNIL, Commission Nationale de l’Informatique et des Libertés) requirements. All patients signed informed consent forms. This study complied with the Declaration of Helsinki. The biocollection affiliated with this cohort was declared to the French Ministry for Higher Education and Research (No. DC-2011-1399) and was validated by the local French Ethics Committee (Comité de Protection des Personnes Ouest IV, Ref 06-15).

### Statistical analysis

Continuous variables are presented as mean±SD or median [first; third quartile] depending on dispersion and categorical variables as count (percentages). Comparisons between preoperative and postoperative values and between PHPT patients and the general French population (SF-36 scores) were performed using Student’s *t* tests. SF-36 score changes after PTX were estimated using linear mixed-effect regression.

Associations between preoperative characteristics [demographic variables, comorbidities (CIRS), surgical indications, biochemical and histopathological findings] and 3-year postoperative PCS and MCS changes were first assessed using a simple linear regression model, with PCS or MCS changes as the dependent variables. No imputation was done in case of missing data. For continuous covariates, potential nonlinearity relations were also tested. A multiple linear regression model was then estimated, including covariates that were statistically significant at the 0.20 threshold (Wald tests) in the simple linear regression models. A backward stepwise procedure was used to select the candidate variables for the final model, which included only the variables statistically significant at the 0.05 level. No correction was applied for multiple testing. Statistical analyses were done using Stata 17.0 software (17.0; StataCorp LLC, College Station, Texas).

## Results

### Baseline patient characteristics

**Figure 1:**
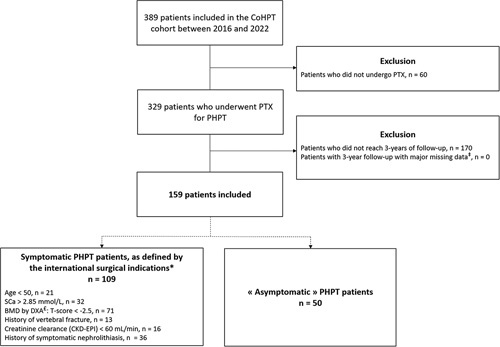
Flow chart. ^‡^Major missing data were BMI, serum calcium, phosphorus, and PTH levels before and 1 year after PTX, and/or SF-36 questionnaire answered before and 3 years after PTX. ^*^Patients can have one or more surgical indications. ^£^At the lumbar spine, left total hip, left femoral neck, and/or distal third of the radius. BMD, bone mineral density; CKD-EPI, Chronic Kidney Disease Epidemiology Collaboration; DXA, dual X-ray absorptiometry; PHPT, primary hyperparathyroidism; PTX, parathyroidectomy, *n*, number of patients; SCa, serum calcium level.

A total of 389 patients were included in the CoHPT cohort between March 2016 and February 2022 (see flow chart, Fig. 1). Among them, 329 patients (84.6%) underwent a standard cervicectomy for PHPT. At the time of the study, 170 patients had been operated on less than 3 years before the time of the study and therefore had not reached the 3-year follow-up. No other patient was excluded for critical missing data. One hundred fifty-nine patients were finally included in the analysis.

Baseline patient characteristics are summarized in Table 1. The mean age at surgery was 62.6±12.7 years and 79.2% were females. The vast majority of patients had a single parathyroid adenoma on histopathologic analysis (83%).

**Table 1:** PHPT patient baseline and histopathological characteristics.

	Patients with PHPT (*n*=159)
Age at PTX, year	62.6 (12.7)
Female gender	126 (79.2)
CIRS score >2 for at least 1 system	6 (3.8)
History of fracture <10 year	40 (25.2)
History of symptomatic nephrolithiasis	36 (22.9)
Fatigue	116 (73.0)
Depression	54 (34.0)
Histopathological results	
Single adenoma	132 (83.0)
Multiple adenomas	8 (5.0)
Hyperplasia	13 (8.2)
Carcinoma	1 (0.6)
No resection or normal gland	5 (3.1)

Continuous data are presented as mean (SDs), and categorical variables as count (percentage).

CIRS indicates cumulative illness rating scale; PHPT, primary hyperparathyroidism; PTX, parathyroidectomy; *n*, number of patients.

### General and biological parameters change after PTX

Three patients experienced postoperative complications (one hematoma and two dysphonia), none requiring reintervention. As expected, PTX was accompanied by a significant decrease in mean SCa (2.43±0.12 vs. 2.74±0.20 mmol/l preoperatively, *P*<0.001), mean urine calcium level (2.52±1.77 vs. 5.07±3.15 mmol/l preoperatively, *P*<0.001), and median serum PTH level (44.1 [35.3–57.6] vs. 96.4 [76.9–126.4] pg/ml preoperatively, *P*<0.001) after 1 year (Table 2). Mean serum phosphorus level significantly increased. A total of 142/155 patients (91.6%) who underwent 6 months of biological evaluation were normocalcemic; three of the four patients who were not evaluated at 6 months (75%) were normocalcemic at the 1-year consultation. Three years after PTX, phosphocalcic parameter improvements were sustained, while a slight but significant increase in BMI and a decrease in eGFR were observed.

**Table 2:** General and phosphocalcic parameters before, 1, and 3 years after PTX in patients with PHPT.

	Before PTX	1 year after PTX	3 years after PTX	*P* value before vs. 1 year	*P* value before vs. 3 years
BMI, kg/m²	26.4 (5.1)	26.6 (5.0)	27.0 (5.3)	0.0689	**0.0155**
Serum calcium, mmol/l	2.74 (0.20)	2.43 (0.12)	2.43 (0.11)	**<0.001**	**<0.001**
Serum phosphorus, mmol/l	0.78 (0.16)	0.98 (0.17)	1.02 (0.16)	**<0.001**	**<0.001**
Serum PTH, pg/ml	96.4 [76.9–126.4]	44.1 [35.3–57.6]	40.5 [31.0–54.4]	**<0.001**	**<0.001**
Serum vitamin D level, ng/ml	25.4 (10.0)	30.5 (9.0)	31.5 (10.0)	**<0.001**	**<0.001**
Urine calcium, mmol/l	5.07 (3.15)	2.52 (1.77)	2.44 (0.11)	**<0.001**	**<0.001**
Serum creatinine, µmol/l	71.82 (22.95)	71.69 (19.83)	73.31 (23.27)	0.934	**0.184**
eGFR (CKD-EPI), ml/min	84.4 (17.7)	83.2 (16.1)	80.5 (17.9)	0.0980	**<0.001**

Data are presented as mean (SDs) or median [first; third quartile] depending on dispersion. Comparisons between preoperative and postoperative values were performed using paired Student’s *t* tests (Wilcoxon matched-pairs signed-rank test for serum PTH). *P* **<**0.05 (bold) was deemed significant.

CKD-EPI indicates Chronic Kidney Disease Epidemiology Collaboration; eGFR, estimated glomerular filtration rate; PTH, parathormone; PTX, parathyroidectomy.

### HR-QoL changes 1 and 3 years after PTX

**Figure 2:**
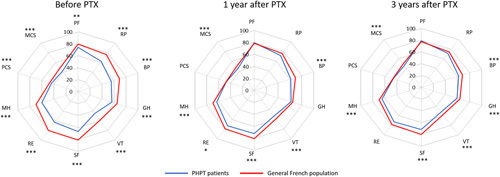
Mean SF-36 questionnaire results in patients with PHPT before, 1, and 3 years after parathyroidectomy, compared with the age-matched and sex-matched general French population. Scores between PHPT patients and age-matched and sex-matched French reference population were compared using Student’s *t* tests; *P<0.05, **P<0.01, and ***P<0.001. BP, bodily pain; GH, general health perception; MCS, mental component score; MH, mental health; PCS, physical component score; PF, physical functioning; PTX, parathyroidectomy; RE, role limitations due to emotional problems; RP, role limitation due to physical problems; SF, social functioning; VT, vitality.

Figure 2 describes the HR-QoL before, 1, and 3 years after PTX for patients with PHPT in comparison with an age-matched and sex-matched general French population. Before surgery, patients with PHPT had impaired HR-QoL in comparison with the reference population as shown by a significantly lower mean PCS (44.6±8.9 vs. 47.6±6.8 in the reference population, *P*<0.001) and MCS (42.3±10.9 vs. 48.9±6.7, *P*<0.001), as well as each dimension score. One year after PTX, PCS, physical functioning, role limitation due to physical problems as well as general health perception became comparable with the general French population, and no significant difference remained. MCS and the other dimension scores remained significantly lower. These improvements were sustained 3 years after PTX except for general health perception, while role limitations due to emotional problems were no longer significantly different.

**Figure 3:**
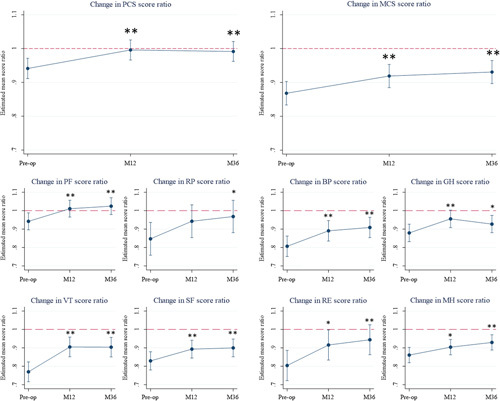
SF-36 scores before, 1, and 3 years after PTX in PHPT patients compared with the age-matched and sex-matched general French population. Mean 1-year and 3-year SF-36 scores changes were estimated using a mixed linear regression model. Values are expressed as the ratios between the scores of patients of the study and the general French population matched for age and sex (red-dashed line). Comparisons between preoperative and postoperative values are the contrasts estimated from the mixed model; *P<0.05 and **P<0.01. BP, bodily pain; GH, general health perception; M12, 1 year after parathyroidectomy; M36, 3 years after parathyroidectomy; MCS, mental component score; MH, mental health; PCS, physical component score; PF, physical functioning; Preop, before parathyroidectomy; RE, role limitations due to emotional problems; RP, role limitation due to physical problems; SF, social functioning; VT, vitality.

In order to compare the 1-year and 3-year postoperative results of the SF-36 questionnaire in PHPT with preoperative values, a linear mixed regression model was used to predict the postoperative changes for each SF-36 score, which were expressed as ratios between the scores of PHPT patients and the general French population (coefficients of the linear mixed effects regression are displayed in Supplementary Table 1, Supplemental Digital Content 1, http://links.lww.com/JS9/A112). After PTX, the mean PCS ratio had increased significantly at the time of the 1-year and 3-year consultation (0.94±0.15 preoperatively vs. 1.00±0.15 at 1 year and 0.99±0.15 at 3 years, *P*<0.01 for both), as well as the MCS ratio (0.87±0.18 preoperatively vs. 0.91±0.18 at 1 year and 0.93±0.17 at 3 years, *P*<0.01 for both), as displayed in Figure 3. This increase at 1 year, which was sustained 3 years after PTX, was also observed for all SF-36 dimension scores except for the role limitation due to physical problems that only improved significantly 3 years after PTX.

### Predictive factors for HR-QoL changes after PTX

**Figure 4:**
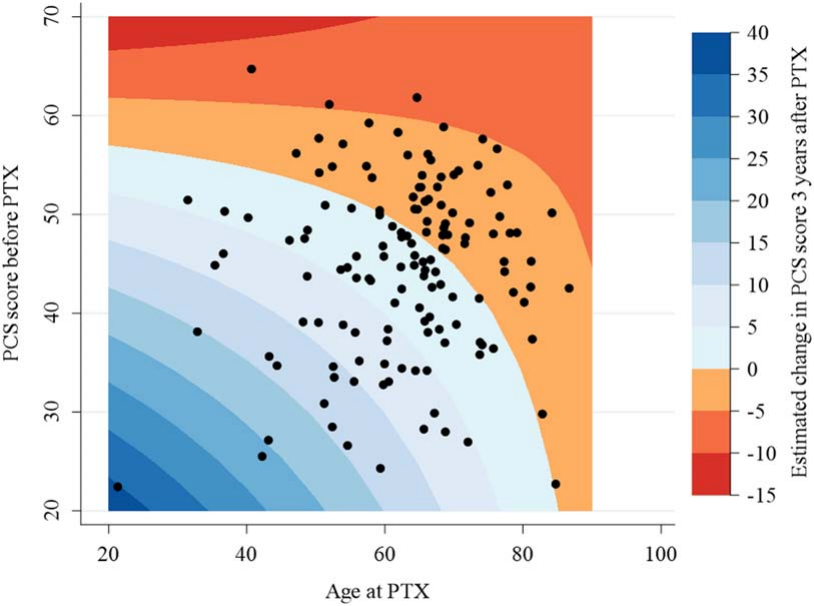
Adjusted predictions of PCS change 3 years after PTX in PHPT patients, according to the age and baseline PCS. PCS change 3 years after PTX according to baseline PCS and age at surgery was predicted using a multiple linear regression analysis. The colored areas are the predicted values; dots form the scatter plot of observed values according to baseline PCS scores and age at surgery. PCS, physical component score; PTX, parathyroidectomy.

Results of simple and multiple linear regression analysis of PCS and MCS changes between baseline and at 3 postoperative years are summarized in Table 3. Among the variables measured before PTX, Table 3, only the PCS score and age were significantly associated with PCS change on multiple regression analysis (*P*<0.001 and *P*=0.016, respectively). Taking into account only the PCS score at baseline and age using a subsequent multivariable regression model (Supplementary Table 2, Supplemental Digital Content 1, http://links.lww.com/JS9/A112) made it possible to explain 41% of the PCS score change 3 years after PTX. From this model, a graphical representation of the predicted PCS score changes 3 years after surgery according to baseline PCS and age at PTX is presented in Figure 4.

**Table 3:** Linear regression analyses of PCS and MCS changes between baseline and 3 postoperative years.

	Univariable linear regression		Multiple regression, after linear selection	
	Coefficient (95% CI)	*P*	Coefficient (95% CI)	*P*
Physical component score				
Baseline PCS(+1 SD)	−0.53 [−0.68 to −0.38]	**<0.001**	−0.46 [−0.60 to −0.31]	**<0.001**
Age, years(+1 SD)	−0.31 [−0.43 to −0.19]	**<0.001**	−0.18 [−0.32 to −0.03]	**0.016**
Sex (female/male)	−2.52 [−6.18 to 1.14]	0.18	−1.10 [−4.49 to 2.29]	0.52
BMI, kg/m²(+1 SD)	0.07 [−0.24 to 0.38]	0.65	—	—
CIRS score >2 for at least 1 system(yes/no)	4.1 [−3.4 to 11.60]	0.28	—	—
History of fracture <10 years(yes/no)	−2.09 [−5.64 to 1.46]	0.25	—	—
History of symptomatic nephrolithiasis(yes/no)	3.32 [−0.12 to 6.77]	0.058	1.07 [−2.13 to 4.28]	0.51
Fatigue(yes/no)	3.61 [0.22–7.01]	**0.037**	1.29 [−1.88 to 4.46]	0.42
Depression(yes/no)	0.34 [−2.74 to 3.42]	0.83	—	—
Mild/classic PHPT^a^(yes/no)	3.31 [−0.48 to 7.09]	0.086	1.54 [−2.05 to 5.13]	0.40
Osteoporosis on DXA^b^(yes/no)	−2.20 [−5.29 to 0.89]	0.16	−0.55 [−3.46 to 2.36]	0.71
SCa, mmol/l(+1 SD)	3.84 [−3.79 to 11.46]	0.32	—	—
Serum phosphorus, mmol/l(+1 SD)	−5.07 [−15.59 to 5.46]	0.34	—	—
Serum PTH, pg/ml(+1 SD)	0.15 [−0.02 to 0.31]	0.080	0.04 [−0.14 to 0.22]	0.66
Serum vitamin D, ng/ml(+1 SD)	−0.31 [−0.60 to −0.02]	**0.037**	0.04 [−0.23 to 0.30]	0.76
Serum vitamin D (squared), ng/ml(+1 SD)	0.002 [0.000–0.004]	0.070	−0.0002 [−0.0019 to 0.0014]	0.77
Urinary calcium, mmol/l(+1 SD)	−0.14 [−0.64 to 0.35]	0.57	—	—
eGFR <60 ml/min (yes/no)	−0.40 [−6.07 to 5.27]	0.89	0.03 [−0.06 to 0.13]	0.52
Histology: no pathological gland was removed (ref.)			—	—
Hyperplasia(yes/no)	−5.10 [−15.89 to 5.68]	0.35	—	—
Single adenoma(yes/no)	−0.31 [−9.44 to 8.81]	0.95	—	—
Multiple adenoma(yes/no)	−6.69 [−18.73 to 5.35]	0.27	—	—
Mental component score				
Baseline MCS(+1 SD)	−0.49 [−0.627 to −0.353]	**<0.001**	−0.43 [−0.61 to 0.25]	**<0.001**
Age, years(+1 SD)	−0.11 [−0.257 to 0.037]	0.140	−0.05 [−0.20 to 0.10]	0.52
Sex (female/male)	−0.06 [−4.225 to 4.106]	0.977	—	—
BMI, kg/m²(+1 SD)	−0.049 [−0.396 to 0.299]	0.783	—	—
CIRS score >2 for at least 1 system(yes/no)	−7.616 [−16.034 to 0.803]	0.076	−5.27 [−13.67 to 3.13]	0.22
History of fracture <10 years(yes/no)	−0.949 [−4.981 to 3.083]	0.642	—	—
History of symptomatic nephrolithiasis(yes/no)	−1.527 [−5.71 to 2.657]	0.472	—	—
Fatigue(yes/no)	4.805 [0.742–8.868]	**0.021**	0.35 [−3.60 to 4.29]	0.86
Depression(yes/no)	3.421 [−0.233 to 7.075]	0.066	−0.03 [−3.59 to 3.53]	0.99
Mild/classic PHPT^a^(yes/no)	0.984 [−3.335 to 5.303]	0.653	—	—
Osteoporosis on DXA^b^(yes/no)	0.721 [−2.797 to 4.238]	0.686	—	—
SCa, mmol/l(+1 SD)	2.895 [−5.743 to 11.533]	0.509	—	—
Serum phosphorus, mmol/l(+1 SD)	2.755 [−9.179 to 14.688]	0.649	—	—
Serum PTH, pg/ml(+1 SD)	0.096 [−0.093 to 0.285]	0.318	—	—
Serum vitamin D, ng/ml(+1 SD)	−0.034 [−0.113 to 0.044]	0.386	—	—
Serum vitamin D (squared), ng/ml(+1 SD)	0.194 [−0.388 to 0.775]	0.511	—	—
Urinary calcium, mmol/l(+1 SD)	5.732 [−0.607 to 12.072]	0.076	4.49 [−1.26 to 10.24]	0.13
eGFR <60 ml/min(yes/no)	9.499 [−2.585 to 21.584]			
Histology: no pathological gland was removed(ref.)	5.453 [−4.771 to 15.677]	0.122	3.97 [−7.53 to 15.48]	0.50
Hyperplasia(yes/no)	12.387 [−1.103 to 25.877]	0.293	0.08 [−9.46 to 9.63]	0.99
Single adenoma(yes/no)	−0.11 [−0.257 to 0.037]	0.072	5.95 [−6.61 to 18.52]	0.35

^a^ Mild PHPT was defined by SCa <2.85 mmol/l.

^b^ Osteoporosis was defined by a *T*-score <−2.5 at lumbar spine, total hip, femoral neck, and/or distal 1/3 radius on DXA.

Association between PCS and MCS change with preoperative variables were tested using a linear regression model. *P*<0.05 (bold) was deemed significant.

CIRS indicates cumulative Illness rating scale; DXA, dual X-ray absorptiometry; eGFR, estimated glomerular filtration rate; MCS, mental component score; PCS, physical component score; PHPT, primary hyperparathyroidism; PTH, parathormone; SCa, serum calcium level.

Multiple regression analysis showed that only the baseline MCS was significantly associated with postoperative MCS change after surgery (*P*<0.001): the lower the preoperative MCS, the greater the postoperative MCS change.

### HR-QoL changes after PTX in asymptomatic patients

Among the 159 included patients, 109 had one or more surgical indications according to the international recommendations^9^: 29 were younger than 50 years, 32 had SCa more than 2.85 mmol/l, 71 had osteoporosis, 13 had history of vertebral fracture, and 16 had history of symptomatic nephrolithiasis (Fig. 1). Fifty patients were therefore considered to truly have asymptomatic PHPT. Their mean age was 64.2±7.4 years, and 40 (80%) were women. Mean SCa, urine calcium, and median PTH decreased significantly 1 year after PTX for these patients, while serum phosphorus increased significantly. These improvements were sustained for 3 years (Supplementary Table 3, Supplemental Digital Content 1, http://links.lww.com/JS9/A112).

**Figure 5:**
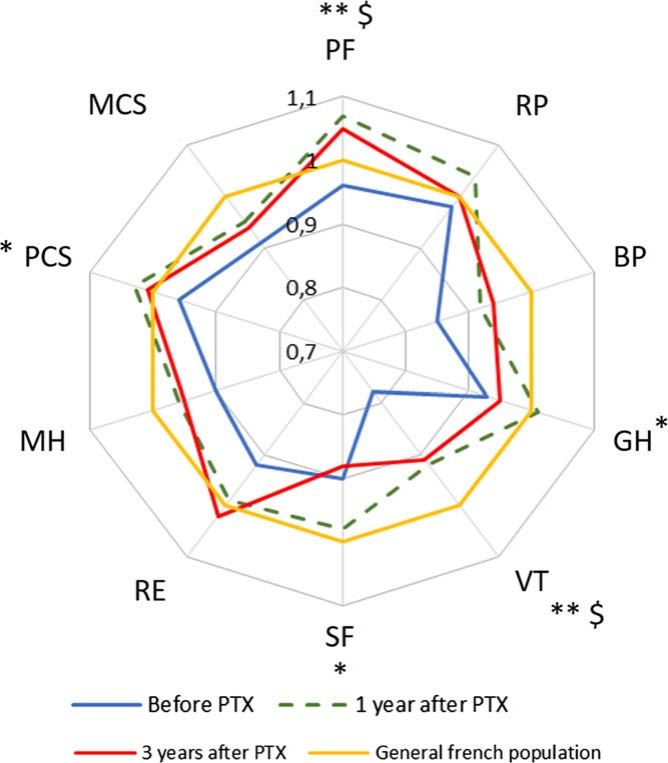
SF-36 questionnaire results before, 1, and 3 years after PTX in asymptomatic PHPT patients. Values are expressed as mean ratios between the scores of patients in the study and the general French population matched for age and sex. Comparisons between preoperative and postoperative values were performed using paired Student’s *t* tests. *P*<0.05 was deemed significant; *P<0.05; **P<0.01, before vs. 1 year after PTX. ^$^ *P*<0.05 before vs. 3 years after PTX. BP, bodily pain; GH, general health perception; M36, 3 years after PTX; MCS, mental component score; MH, mental health; PCS, physical component score; PF, physical functioning; RE, role limitations due to emotional problems; RP, role limitation due to physical problems; SF, social functioning; VT, vitality.

Figure 5 shows the SF-36 questionnaire results in asymptomatic PHPT patients before and after PTX. One year after PTX, a significant increase was observed in PCS (0.95 [95% CI 0.89; 1.01] preoperatively vs. 1.02 [0.98−1.08] postoperatively, *P*=0.012) as well as in physical functioning, general health perception, vitality, and social functioning dimension scores. The MCS, as well as the other scores, were not significantly modified. Three years after PTX, physical functioning (1.05±0.04 vs. 0.96±0.04 preoperatively, *P*=0.023) and vitality (0.78±0.05 vs. 0.91±0.05 preoperatively, *P*=0.012) scores remained significantly increased. None of the scores decreased significantly during follow-up.

## Discussion

In this prospective monocentric observational study, SF-36 component scores PCS and MCS as well as all dimension scores increased significantly 1 and/or 3 years after PTX. PCS reached that of an age-matched and sex-matched reference population. PCS positive change 3 years after PTX was mainly associated with lower baseline PCS and younger age on multivariable analysis, while only baseline MCS was associated with postoperative MCS change. Finally, in patients with asymptomatic PHPT, physical functioning and vitality increased significantly 3 years after PTX.

PHPT is known to be associated with impaired HR-QoL in comparison with the general population^8^,^16^. In line with these observations, both PCS and MCS, as well as all SF-36 dimension scores, were significantly decreased in PHPT patients before surgery in comparison with a French age-matched and sex-matched general population in the present study. Interestingly, the deleterious impact of PHPT appeared to be greater on mental health (through MCS). In accordance with these results, Weber *et al*.^16^ found that 191 patients with PHPT had a reduction in both PCS and MCS, but who presented stronger MCS impairement in comparison with the healthy German population (PCS 42.66±11.30 vs. 50.21±10.24, and MCS 41.21±13.48 vs. 51.54±8.14, *P*<0.001 for both). In 191 patients with asymptomatic PHPT, Bollerslev *et al*.^24^ found HR-QoL impairment predominating on psychological domains and MCS in comparison with Swedish normative data (MCS 46.3±12.4 in PHPT patients vs. 51.0±10.4, *P*<0.05). Although the mechanism by which PHPT causes nonspecific symptoms and impairs HR-QoL is not perfectly understood, a direct effect of PTH on the central nervous system through its abundant receptors in the brain^35^ could participate in this phenomenon.

The main finding of the present study is that HR-QoL was significantly improved and sustained 3 years after PTX in a population of patients with PHPT, regardless of severity. PCS, MCS, and all SF-36 dimension scores increased significantly. Mean postoperative PCS and MCS increased by 5 and 6%, respectively, 3 years after PTX. A 5% change in SF-36 items is the lowest that can be considered as a relevant clinical change^36^, suggesting that this improvement is of clinical significance. The question of HR-QoL after PTX for PHPT is still a matter of controversy. On the one hand, short-term studies, mostly observational, have suggested an improvement within the first 15 months after PTX using the SF-36 questionnaire^12^,^17^,^37^,^38^. The largest study comes from Weber *et al*.^16^ who compared 191 patients with PHPT with 186 thyroidectomized controls and found that both PCS and MCS increased significantly 12 months after PTX. However, it should be noted that patients who are prescribed surgery could have high expectations for improvement, which could lead to a placebo effect^24^,^39^. This could interfere with HR-QoL evaluation if performed too early and explain the reason postoperative improvement in HR-QoL has been found as early as 3 months after PTX^15^. By showing that early improvement is observed 3 years after PTX, the present study makes it possible to avoid this potential bias.

On the other hand, studies with longer follow-ups, including randomized trials, have reported only modest improvement, especially in comparison with the management of PHPT without surgery^21^,^25^. The largest randomized study compared 89 patients who underwent PTX vs. 90 without surgery^25^. After 10 years of follow-up, only vitality and social functioning were modestly improved after PTX, whereas SF-36 results remained stable during observation. In contrast to the present study, those studies only included patients with asymptomatic PHPT. In accordance with these findings, focusing on patients with asymptomatic PHPT in our cohort resulted only in a significant improvement of two dimension scores (physical functioning and vitality) 3 years after surgery. Even if they are significant, these results suggest that asymptomatic patients, that is patients who do not have surgical indications according to current guidelines^9^, could benefit less from surgery vis-à-vis HR-QOL. However, this assumption should be noted with caution since our study could be underpowered to detect significant differences in other SF-36 scores in asymptomatic patients.

Preoperatively, only lower baseline PCS and younger age at surgery were found to be significantly associated with improvement in PCS 3 years after surgery in multivariable analysis. A multivariable model including these two parameters explained 41% of the postoperative PCS change. The fact that younger patients are more likely to benefit from PTX in terms of HR-QoL is in accordance with a study that found that patients younger than 70 years presented more significant improvement^15^. On the other hand, the present study failed to identify factors other than baseline MCS to be significantly associated with postoperative MCS change. It could therefore be hypothesized that postoperative MCS evolution could be predicted by a combination of symptoms such as the Pasieka Illness Questionnaire^5^ (not validated in French) instead of isolated symptoms such as asthenia and depression, which were chosen in the present study because they are frequent in PHPT and likely to improve after PTX^5^,^6^,^14^. As previously shown^13^, SCa and serum PTH levels were not significantly associated with HR-QoL change. As reported by Pretorius *et al*.^25^, baseline SCa could be associated with SF-36 dimension scores change rather than PCS and MCS, showing the complexity of HR-QoL impairment during PHPT.

This study has some limitations that should be noted. First, HR-QoL evolution after PTX was not compared with a control group of patients with PHPT without surgery. The spontaneous evolution of HR-QoL in asymptomatic PHPT has been previously suggested to worsen during a 3-year follow-up^21^ or be stable for 10 years^25^. Secondly, HR-QoL was only assessed using the SF-36 questionnaire, which is not specific to PHPT. The PHPQoL has been proposed as a validated questionnaire adapted for PHPT^40^,^41^ but is not validated in French. Other validated tools have been widely used to evaluate HR-QoL in PHPT, such as patient Health Questionnaire-9 (for depression), the Hospital Anxiety and Depression Scale^16^, the 15D instrument^18^,^22^, or other validated HR-QoL uni-scales^5^. The SF-36 questionnaire has the advantage of being validated in French and enables comparisons with the literature. Thirdly, as it has not been performed systematically in our center during the inclusion period, missing data regarding 24-h urine calcium excretion prevented us to take this information into account when defining asymptomatic patients. Despite these limits, this study is the largest to describe HR-QoL change in PHPT patients 3 years after PTX, thereby making a comparison with an age-matched and sex-matched reference healthy population possible.

## Conclusion

In this prospective monocentric observational study, both physical and mental dimensions of HR-QoL (SF-36 PCS and MCS) improved significantly 1 and 3 years after PTX in patients with PHPT. Three years after PTX, SF-36 PCS was not significantly different from that of an age-matched and sex-matched reference healthy population, while MCS remained lower. In a subgroup of patients with asymptomatic PHPT, two-dimension scores of the SF-36 questionnaire improved significantly. These results suggest that the short-term benefit of PTX on HR-QoL in patients with PHPT is maintained over time.

## Patient consent

All patients gave their informed consent.

## Sources of funding

This work was supported by the Appel d’Offre Interne (AOI) of CHU Nantes (#AOI-CHUN-2015-CO and RC19_0415_1).

## Author contribution

S.F.: contributed to the conception and design of the study, analysis and interpretation of data, and first drafting of the article; B.P. and E.M.: contributed to the conception and design of the study, analysis and interpretation of data, and revising the draft critically for important intellectual content; C.C., M.L.B., and M.G.: contributed to the acquisition of data and revising the draft critically for important intellectual content; C.B. and B.C.: contributed to the conception and design of the study and revising the draft critically for important intellectual content; M.W.: contributed to the analysis and interpretation of data and revising the draft critically for important intellectual content. All authors approved the final version of the article.

## Conflicts of interest disclosure

The authors have no conflicts of interest to disclose.

## Research registration unique identifying number (UIN)

1. Name of the registry: Clinical trial.
2. Unique identifying number or registration ID: NCT05469087.
3. Hyperlink to your specific registration (must be publicly accessible and will be checked): https://clinicaltrials.gov/ct2/show/NCT05469087

## Guarantor

Prof Eric Mirallié.

## Data availability

Data are available on request to the corresponding author.

## Provenance and peer review

Not commissioned, externally peer-reviewed.

## Supplementary Material
