# Supplementary material for: Parathyroidectomy for primary hyperparathyroidism: effect on quality of life after 3 years – a prospective cohort study
Source: Int J Surg. 2023 Mar 24;109(3):364–73. doi: 10.1097/JS9.0000000000000282 (PMC10389353; doi:10.1097/JS9.0000000000000282)
Supplement: Supplementary file 1 [file js9-109-364-s001.docx]

**Supplementary Table 1. Coefficients of the linear mixed effects regression of SF-36 score-ratios on time.**

| SF-36 score-ratio | **Visit (reference = Pre-op)** | **Coefficient [95% CI]** | **P-value** |
| --- | --- | --- | --- |
| Physical component score | 1 year | 0.055 [0.026–0.083] | **<0.001** |
|  | 3 years | 0.05 [0.022–0.079] | **0.001** |
|  | intercept | 0.941 [0.911–0.971] | **<0.001** |
| Mental component score | 1 year | 0.069 [0.032–0.106] | **<0.001** |
|  | 3 years | 0.082 [0.045–0.119] | **<0.001** |
|  | intercept | 0.942 [0.896–0.988] | **<0.001** |
| PF | 1 year | 0.069 [0.032–0.106] | **<0.001** |
|  | 3 years | 0.082 [0.045–0.119] | **<0.001** |
|  | intercept | 0.942 [0.896–0.988] | **<0.001** |
| RP | 1 year | 0.096 [-0.006–0.197] | **0.064** |
|  | 3 years | 0.121 [0.021–0.222] | **0.018** |
|  | intercept | 0.847 [0.758–0.936] | **<0.001** |
| BP | 1 year | 0.084 [0.028–0.14] | **0.003** |
|  | 3 years | 0.102 [0.046–0.158] | **<0.001** |
|  | intercept | 0.807 [0.751–0.862] | **<0.001** |
| GH | 1 year | 0.076 [0.038–0.114] | **<0.001** |
|  | 3 years | 0.048 [0.01–0.085] | **0.013** |
|  | intercept | 0.879 [0.832–0.927] | **<0.001** |
| VT | 1 year | 0.135 [0.086–0.184] | **<0.001** |
|  | 3 years | 0.134 [0.085–0.183] | **<0.001** |
|  | intercept | 0.77 [0.716–0.824] | **<0.001** |
| SF | 1 year | 0.064 [0.017–0.111] | **0.008** |
|  | 3 years | 0.07 [0.023–0.117] | **0.003** |
|  | intercept | 0.829 [0.78–0.879] | **<0.001** |
| MH | 1 year | 0.043 [0.005–0.081] | **0.026** |
|  | 3 years | 0.069 [0.031–0.106] | **<0.001** |
|  | intercept | 0.861 [0.819–0.903] | **<0.001** |
| RE | 1 year | 0.111 [0.025–0.198] | **0.012** |
|  | 3 years | 0.14 [0.053–0.226] | **0.001** |
|  | intercept | 0.804 [0.722–0.886] | **<0.001** |

The linear mixed effects regression model was applied on HR-QoL component at each date after surgery using the pre-operative value as reference. Statistical significance of regression coefficients was tested with Wald tests. P < 0.05 (**bold**) was deemed statistically significant.

**CI:** confidence interval**, PTX:** parathyroidectomy, **Pre-op:** before PTX, **M12:** 1 year after PTX, **M36:** 3 years after PTX, **PCS**: physical component score, **MCS**: mental component score, **PF**: physical functioning, **RP**: Role limitation due to physical problems, **BP**: Bodily pain, **GH**: General health perception, **VT**: vitality, **SF**: social functioning, **RE**: role limitations due to emotional problems, **MH**: mental health.

**Supplementary table 2. Multiple linear regression analyses of PCS change 3 years after PTX according to baseline PCS and age at surgery**

|  | **Coefficient (95% CI)** | **P-value** |
| --- | --- | --- |
| **Baseline PCS** | -1.32 [-1.89 – -0.74] | **<0.001** |
| **Age at PTX** | -0.87 [-1.28 – -0.45] | **<0.001** |
| **Interaction** | 0.01 [0.00 – 0.02] | **0.005** |

P < 0.05 (**bold**) was deemed significant.

**PCS:** physical component score, **PTX:** parathyroidectomy, **95%CI:** 95% confidence interval.

**Supplementary table 3. Asymptomatic PHPT patient characteristics before and at 1 and 3 post-operative years**

|  | **Before PTX** | **1 year after PTX** | | **3 years after PTX** | **P-value**  **Before vs. 1-year** | **P-value**  **Before vs. 3-years** |
| --- | --- | --- | --- | --- | --- | --- |
| **Age at PTX, year** | 64.2 (7.4) | - | - | | - | - |
| **Female gender** | 40 (80.0) | - | - | | - | - |
| **BMI, kg/m²** | 27.7 (5.8) | 27.5 (5.4) | | 27.8 (5.5) | 0.46 | 0.71 |
| **Serum calcium, mmol/L** | 2.70 (0.09) | 2.41 (0.08) | | 2.43 (0.09) | **<0.0010** | **<0.0010** |
| **Serum phosphorus, mmol/L** | 0.80 (0.13) | 1.00 (0.18) | | 1.04 (0.17) | **<0.0010** | **<0.0010** |
| **Serum PTH, pg/mL** | 85.0 [76.1–105.6] | 47.1 [37.5–55.0] | | 40.7 [33.4–54.5] | **<0.0010** | **<0.0010** |
| **Serum vitamin D level, ng/mL** | 25.6 (7.8) | 30.5 (8.5) | | 31.3 (8.9) | **<0.0011** | **<0.0010** |
| **Urine calcium, mmol/L** | 5.48 (3.32) | 2.51 (1.77) | | 2.64 (1.79) | **<0.0010** | **<0.0011** |
| **Serum creatinine, µmol/L** | 66.6 (12.3) | 67.3 (1.80) | | 67.7 (13.3) | 0.67 | 0.48 |
| **eGFR, mL/min** | 86.2 (11.4) | 85.6 (112) | | 82.3 (16.4) | 0.30 | **0.034** |

Comparisons between pre- and post-operative values were performed using paired Student's *t*-tests. P < 0.05 (**bold**) was deemed significant.

**PTX**: parathyroidectomy, **BMI**: body mass index, **PTH**: parathormone, **eGFR**: estimated glomerular filtration rate.
